# COVID-19 vaccine effectiveness in patients with lung cancer: challenges in the Omicron era

**DOI:** 10.1186/s12879-026-12741-9

**Published:** 2026-02-06

**Authors:** Seyed M. Hosseini-Moghaddam, Sarah Swayze, Jeffrey C. Kwong, Frances A. Shepherd

**Affiliations:** 1 ICES, Toronto, ON Canada; 2 Transplant-Oncology Infectious Diseases, University Health Network, Toronto, ON Canada; 3 Division of Infectious Diseases, Department of Medicine, University of Toronto, Toronto, ON Canada; 4 Divisions of Medical Oncology and Hematology, Department of Medicine, University of Toronto, Toronto, ON Canada; 5 Princess Margaret Cancer Centre, University Health Network, 700 University Ave, Toronto, M5G 1Z5 ON Canada; 6 Public Health Ontario, Toronto, ON Canada; 7 Department of Family and Community Medicine, University of Toronto, Toronto, ON Canada; 8 Dalla Lana School of Public Health, University of Toronto, Toronto, ON Canada; 9 Centre for Vaccine Preventable Diseases, University of Toronto, Toronto, ON Canada; 10 Ajmera Transplant Centre, University Health Network, University of Toronto, 9Ma RS-9078, 585 University Ave, Toronto, Canada

**Keywords:** SARS-CoV-2, COVID-19, Vaccine effectiveness, Lung cancer, mesothelioma

## Abstract

**Background:**

During the Omicron era, COVID-19 vaccines demonstrated over 90% effectiveness against severe disease in the general population. However, vaccine effectiveness (VE) in patients with lung cancer, who are at high risk for severe COVID-19, has not been well characterized.

**Methods:**

In this population-based cohort, we employed a test-negative design using linked data from the Ontario Cancer Registry, health administrative databases, and the Ontario vaccination registry. We included all patients with active lung cancer or mesothelioma residing in Ontario, Canada, who were symptomatic and underwent SARS-CoV-2 RT-PCR testing from January 2, 2022, to June 30, 2025. The outcome of interest was a severe outcome, defined as hospitalization within 14 days or death within 30 days following SARS-CoV-2 test. We constructed an analytic cohort that included the earliest testing episode among test-positive and test-negative patients with severe outcomes. We also performed a sensitivity analysis comparing test-positive patients with severe outcomes to randomly selected testing episodes among test-negative patients.

**Results:**

Among 47,344 tested patients, 3,332 (7.0%) were SARS-CoV-2 test-positive, of whom 1,967 (59%) experienced severe outcomes; 16,257 test-negative patients also developed severe outcomes. Adjusted VE against severe COVID-19 was 52% (95% CI: 42%, 60%) within 7–179 days post-vaccination, declining to 16% (95%CI: 0%, 30%) thereafter. In sensitivity analyses comparing 1,967 test-positive patients with severe outcomes to 18,922 randomly selected test-negative patients, adjusted VE was 49% (95% CI: 39%, 58%), declining to 12% (95% CI: −4% 26%) thereafter.

**Conclusions:**

In this large population-based study of patients with lung cancer and mesothelioma, COVID-19 vaccination was associated with moderate protection against severe outcomes, including hospitalization and death. These findings emphasize the importance of optimizing alternative preventive strategies, such as masking, social distancing, ensuring household and caregiver vaccinations, and improving post-infection management through early antiviral therapy and close monitoring for severe disease.

**Clinical trial number:**

Not applicable.

**Supplementary information:**

The online version contains supplementary material available at 10.1186/s12879-026-12741-9.

## Background

Lung cancer remains the leading cause of cancer-related mortality worldwide, affecting more than 2 million individuals annually [1]. Since the onset of the COVID-19 pandemic, different studies have shown that patients with lung cancer are at significantly increased risk of severe COVID-19 [2–5]. The Thoracic Cancers International COVID-19 Collaboration (TERAVOLT) global consortium, which focuses on patients with thoracic malignancies, reported approximately 30% mortality among patients with lung cancer who developed COVID-19 during the early pandemic period, with subsequent analyses confirming persistently higher risks of severe outcomes in this population compared with other cancer types [6–8]. A nationwide French study of more than 35 million individuals also showed that patients with lung cancer are at a three-fold higher risk of hospitalization and ICU admission following COVID-19 diagnosis compared to the general population [9]. In a provincial cohort study in Canada, patients with lung cancer had the highest risk of mortality among all patients with solid tumors following COVID-19 diagnosis [10].

A recent large-scale provincial study conducted in the general population of Ontario estimated vaccine effectiveness (VE) of 98% (95% CI: 88%, 100%) against severe outcomes (hospitalization or death) [11]. Similarly, a population-based study estimated a VE of 94.5% against COVID-19-associated death in patients with cancer [12]. However, these studies did not evaluate VE in high-risk populations such as patients with lung cancer.

The Omicron variant is associated with a lower mortality risk compared to earlier variants. A recent multinational cohort study reported 28-day mortality rates of 14% for wild-type SARS-CoV-2, 10% for Alpha, 9% for Delta, and 6% for Omicron variants [13]. In Ontario, Omicron emerged in November 2021 and became the dominant strain ( > 97% of sequenced cases) by January 2022 [14]. Since its emergence, COVID-19 vaccination rates have declined, likely reflecting a reduced public and clinical perception of risk, particularly following the emergence of milder SARS-CoV-2 variants, and the limited availability of vaccine effectiveness data in high-risk populations, which may contribute to uncertainty and lower vaccine uptake among vulnerable patients [15–21].

To date, no study has specifically assessed COVID-19 vaccine effectiveness in patients with lung cancer during the Omicron era, a period in which vaccination uptake in the general population has been suboptimal, with particularly poor booster coverage over the past two seasons [22]. In this study, we aimed to evaluate the effectiveness of COVID-19 vaccination in reducing the risk of severe COVID-19 outcomes among patients with lung cancer.

## Materials and methods

### Study design

We conducted a province-wide test-negative–design study, analogous to a nested case–control design, within a cohort of patients with lung cancer or mesothelioma who underwent SARS-CoV-2 testing and developed severe outcomes. In this framework, both cases and controls are drawn from a symptomatically tested population, consistent with the core principle of the test-negative design, in which individuals are tested for the same clinical indication to detect the pathogen of interest [23–26].

The primary analytic cohort included the earliest testing episode associated with severe outcomes among both test-positive and test-negative patients, with cases defined as test-positive individuals who experienced hospitalization or death and controls as symptomatic test-negative individuals with severe outcomes. A sensitivity analysis compared the earliest testing episode among test-positive patients with severe outcomes to randomly selected testing episodes among test-negative patients.

We adhered to the Strengthening the Reporting of Observational Studies in Epidemiology (STROBE) guideline for reporting (Appendix, Supplemental Table 1).

### Data sources and definitions

We utilized the Ontario Cancer Registry (OCR) to identify individuals with active lung cancer and mesothelioma. Through a unique encoded identifier for each individual, we linked OCR data with provincial databases on SARS-CoV-2 laboratory testing, COVID-19 vaccination, and health administrative records. Definitions and data sources for variables and comorbidities are detailed in Appendix Supplemental Table 2. Data analysis was conducted at ICES, a not-for-profit research institute in Ontario, Canada. The use and analysis of this data were authorized under Section 45 of Ontario’s Personal Health Information Protection Act, which does not require approval from an ethics board.

### Study population

We included all Ontarians aged ≥18 years with active lung cancer or mesothelioma diagnosed before December 31, 2024, who were eligible for provincial health insurance and underwent SARS-CoV-2 RT-PCR testing from January 2, 2022, to June 30, 2025. We excluded those with a positive SARS-CoV-2 test within the preceding 60 days or who received any non-Health Canada-authorized COVID-19 vaccines.

### Exposure and outcomes

COVID-19 vaccination status was identified using the COVaxON database, a centralized provincial registry that records real-time vaccination data, including vaccine product, administration date, and dose number. Vaccination status for each individual was determined as of their index date (i.e., the date of SARS-CoV-2 testing).

The outcome of interest was severe COVID-19, defined as COVID-19-related hospitalizations or death. Hospitalization was considered COVID-19-related if an RT-PCR-confirmed SARS-CoV-2 infection occurred within 14 days before or three days after hospital admission, as recorded in the provincial COVID-19 reportable disease surveillance system (Public Health Case and Contact Management Solution; CCM). We determined all-cause mortality associated with an RT-PCR-confirmed SARS-CoV-2 infection occurring within 30 days before death or seven days post-mortem or fatal outcome recorded in CCM. If cases had multiple positive test results, the date of the first positive test was used as the index date. For test-negative controls, one symptomatic testing episode was randomly selected during the observation period to ensure comparability and maintain analytic feasibility. If individuals in the control group had more than one negative test result, a single negative test was randomly chosen as their index date.

### Covariates

We obtained information on age, sex, comorbidity burden (as measured by the Charlson-Deyo Comorbidity index, a validated tool that quantifies comorbid conditions to predict mortality risk) and residential postal code from the Registered Persons Database (RPDB) [27]. Residential postal codes were used to determine each individual’s public health unit of residence. Socioeconomic status was assessed using the nearest census-based neighbourhood income quintile from the 2016 Canadian Census.

We included receipt of influenza vaccination during the previous two influenza seasons as a proxy for health behaviours, using physician and pharmacist billing claims in the Ontario Health Insurance Plan and Ontario Drug Benefit databases, respectively.

### Statistical analysis

Descriptive analyses were performed to compare baseline characteristics between cases and controls, as well as between vaccinated (7–179 days before testing) and unvaccinated individuals.

VE was estimated as VE = (1 − odds ratio) × 100, using multivariable logistic regression models to compare the odds of vaccination between cases and controls. VE was assessed for 14-day hospitalization, 30-day mortality, and a composite severe outcome defined as hospitalization within 14 days or death within 30 days of the index test. Vaccination status was modeled as a categorical exposure variable based on the interval since the most recent vaccine dose; individuals vaccinated 7–179 days before the index date were classified as vaccinated, whereas those without vaccination during this period were considered unvaccinated.

VE was estimated for a composite severe outcome, defined as hospitalization and/or death. Models were adjusted for age, sex, comorbidity status (Charlson–Deyo score), calendar time, public health region, area-level socioeconomic indicators (occupation, income, household size, and proportion of visible minorities), neighbourhood income quintile, influenza vaccination status, and prior SARS-CoV-2 infection occurring ≥90 days before the index date. Both unadjusted and adjusted odds ratios (ORs) were reported, accounting for variables previously associated with severe COVID-19 outcomes or vaccination status (i.e., potential confounders). The number of prior vaccine doses was not included as an independent covariate because it is highly correlated with time since vaccination and age, both of which were incorporated into the multivariable models.

To further evaluate vaccine performance, we estimated both seasonal and relative VE using the same analytic framework. For seasonal VE, individuals who had not received a COVID-19 vaccine during the corresponding vaccination season, defined as those unvaccinated or whose most recent dose was administered ≥180 days before the index test, were classified as unvaccinated. For relative VE, analyses were restricted to vaccinated individuals only, with those whose most recent vaccine dose was given ≥180 days prior serving as the reference group. This approach enabled assessment of waning protection over time since the most recent vaccination, independent of the unvaccinated population.

All analyses were conducted using SAS software, version 9.4 (SAS Institute, Cary, NC, USA). Statistical tests were two-sided, and *p* < 0.05 was considered statistically significant.

## Results

**Fig. 1:**
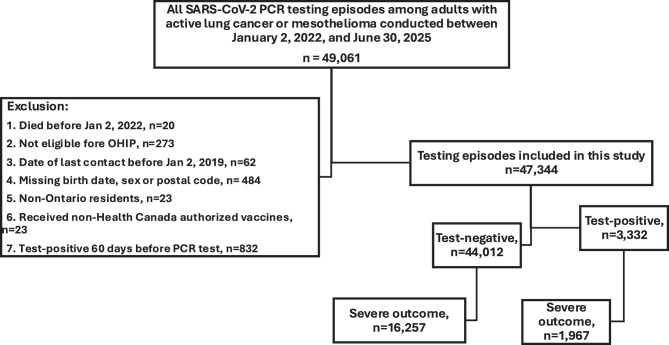
Study flowchart, study cohort

Overall, 49,061 patients with lung cancer or mesothelioma underwent SARS-CoV-2 testing from January 2, 2022, to June 30, 2025. After excluding 1,717 individuals who met one or more exclusion criteria, 47,344 testing episodes remained, including 3,332 test-positive and 44,012 test-negative patients (Fig. 1). Among test-positive patients, 1,967 experienced severe outcomes and were compared with 16,257 test-negative patients with severe outcomes; patient characteristics for the primary study cohort are shown in Table 1. We compared patient characteristics among 1,300 individuals who were unvaccinated at the time of SARS-CoV-2 testing, 7,045 patients who were tested 7–179 days after vaccination, and 9,879 who were tested ≥180 days post-vaccination (Table 2). In this cohort, 3189 (19.6%) test-negative patients and 623 (31.7%) test-positive patients died (*p* < 0.001). Hospitalization without death occurred in 13,068 (80.4%) test-negative patients and 1344 (68.3%) test-positive patients (*p* < 0.001). In adjusted analyses, VE against severe COVID-19 was 52% (95% CI: 42%, 60%), decreasing to 16% (95% CI: 0%, 30%) among individuals ≥180 days after their most recent vaccine dose (Table 3). Adjusted seasonal VE was 44% (95% CI: 37%, 50%), and adjusted relative VE against severe COVID-19 was 42% (95% CI: 35%, 49%).

**Table 1:** Comparison of test-negative and test-positive patients with severe outcomes

Variable	Test-negative *N* = 16,257	Severe COVID-19 *N* = 1,967	P-Value
Age (year), mean ± SD	70.34 ± 10.02	71.48 ± 10.09	< 0.001
Sex F M	7,992 (49.2%) 8,265 (50.8%)	915 (46.5%) 1,052 (53.5%)	0.027
PHU region* Central East Central West Durham Eastern Northern Ottawa Peel South West Toronto York Missing	1,552 (9.5%) 2,962 (18.2%) 799 (4.9%) 1,536 (9.4%) 1,560 (9.6%) 930 (5.7%) 1,000 (6.2%) 1,960 (12.1%) 2,646 (16.3%) 1,100 (6.8%) 212 (1.3%)	148 (7.5%) 374 (19.0%) 73 (3.7%) 149 (7.6%) 197 (10.0%) 119 (6.0%) 146 (7.4%) 2,271 (12.0%) 354 (18.0%) 138 (7.0%) 20 (1.0%)	< 0.001
Living in rural areas	2,345 (14.4%)	221 (11.2%)	< 0.001
Neighborhood income quintile * 1 2 3 4 5	4,247 (26.1%) 3,716 (22.9%) 2,990 (18.4%) 2,736 (16.8%) 2,539 (15.6%)	535 (27.2%) 445 (22.6%) 365 (18.6%) 314 (16.0%) 305 (15.5%)	0.888
Vaccine doses prior to PCR test 0 1 2 3 4 5 6 7 ≥8	1,013 (6.2%) 163 (1.0%) 2,484 (15.3%) 6,072 (37.4%) 393 (20.0%) 1,963 (12.1%) 916 (5.6%) 378 (2.3%) 114 (0.7%)	166 (8.4%) 27 (1.4%) 334 (17.0%) 697 (35.4%) 393 (20.0%) 213 (10.8%) 83 (4.2%) 45 (2.3%) 9 (0.4%)	< 0.001
Vaccination Status at PCR test date Unvaccinated 7–179 days post- vaccination ≥180 days post-vaccination	1,122 (6.9%) 6,449 (39.7%) 8,686 (53.4%)	178 (9.0%) 596 (30.3%) 1,193 (60.7%)	< 0.001
Month of SARS-CoV-2 PCR test Jan-22 Feb-22 Mar-22 Apr-22 May-22 Jun-22 Jul-22 Aug-22 Sep-22 Oct-22 Nov-22 Dec-22 Jan-23 Feb-23 Mar-23 Apr-23 May-23 June 23 Jul-23 Aug-23 Sep-23 Oct-23 Nov-23 Dec-23 Jan-24 Feb-24 Mar-24 Apr-24 May-24 Jun-24 Jul-24 Aug-24 Sep-24 Oct-24 Nov-24 Dec-24 Jan-25 Feb-25 Mar-Jun 25	936 (5.8%) 774 (4.8%) 809 (5.0%) 686 (4.2%) 640 (3.9%) 600 (3.7%) 507 (3.1%) 547 (3.4%) 553 (3.4%) 547 (3.4%) 562 (3.5%) 499 (3.1%) 515 (3.2%) 497 (3.1%) 523 (3.2%) 429 (2.6%) 358 (2.2%) 276 (1.7%) 265 (1.6%) 301 (1.9%) 284 (1.7%) 361 (2.2%) 359 (2.2%) 360 (2.2%) 404 (2.5%) 363 (2.2%) 352 (2.2%) 366 (2.3%) 364 (2.2%) 294 (1.8%) 320 (2.0%) 289 (1.8%) 260 (1.6%) 169 (1.0%) 153 (0.9%) 158 (1.0%) 147 (0.9%) 138 (0.8%) 292 (1.8%)	159 (8.1%) 63 (3.2%) 50 (2.5%) 117 (5.9%) 81 (4.1%) 32 (1.6%) 72 (3.7%) 66 (3.4%) 80 (4.1%) 102 (5.2%) 85 (4.3%) 91 (4.6%) 82 (4.2%) 54 (2.7%) 68 (3.5%) 38 (1.9%) 26 (1.3%) 14 (0.7%) 17 (0.9%) 24 (1.2%) 38 (1.9%) 62 (3.2%) 72 (3.7%) 79 (4.0%) 75 (3.8%) 24 (1.2%) 9 (0.5%) 12 (0.6%) 16 (0.8%) 19 (1.0%) 32 (1.6%) 36 (1.8%) 37 (1.9%) 36 (1.8%) 30 (1.5%) 25 (1.3%) 23 (1.2%) 11 (0.6%) 10 (0.5%)	< 0.001
Influenza vaccination, 2 recent seasons	4,565 (28.1%)	560 (28.5%)	0.717
Comorbidities Previous TIA/stroke Asthma Previous Cardiac ischemia Congestive heart failure Chronic kidney disease COPD Dementia Diabetes mellitus Hypertension Immunocompromised ** Liver disease or cirrhosis	657 (4.0%) 3,050 (18.8%) 2,523 (15.5%) 2,540 (15.6%) 2,435 (15.0%) 5,812 (35.8%) 702 (4.3%) 5,183 (31.9%) 11,297 (69.5%) 128 (0.8%) 731 (4.5%)	104 (5.3%) 397 (20.2%) 354 (18.0%) 401 (20.4%) 382 (19.4%) 769 (39.1%) 117 (5.9%) 713 (36.2%) 1,449 (73.7%) 21 (1.1%) 106 (5.4%)	0.009 0.128 0.004 < 0.001 < 0.001 0.004 < 0.001 < 0.001 < 0.001 0.192 0.074
Frailty score, Mean ± SD	3.05 ± 5.05	5.59 ± 6.79	< 0.001
Charlson-Deyo Comorbidity index, Mean ± SD	0.97 ± 1.34	1.24 ± 1.48	< 0.001

COPD: Chronic obstructive pulmonary disease, SD: standard deviation * Missing < 0.3% **HIV infection, Organ transplant

**Table 2:** Comparison of unvaccinated during the season and individuals 7–179 and ≥180 days post- vaccination

Variable	Unvaccinated, *N* = 1,300	7–179 days post- vaccination, *N* = 7,045	≥180 days post- vaccination, *N* = 9,879	P-value
Age (year), mean ± SD	66.6 ± 10.8	72.5 ± 9.3	69.5 ± 10.1	< 0.001
Sex F M	606 (46.6%) 694 (53.4%)	3,459 (49.1%) 3,586 (50.9%)	4,842 (49.0%) 5,037 (51.0%)	0.291
PHU region* Central East Central West Durham Eastern Northern Ottawa Peel South West Toronto York	130 (10.0%) 240 (18.5%) 50 (3.8%) 123 (9.5%) 127 (9.8%) 61 (4.7%) 86 (6.6%) 175 (13.5%) 213 (16.4%) 81 (6.2%)	654 (9.3%) 1,263 (17.9%) 341 (4.8%) 694 (9.9%) 645 (9.2%) 457 (6.5%) 393 (5.6%) 902 (12.8%) 1,106 (15.7%) 483 (6.9%)	916 (9.3%) 1,833 (18.6%) 481 (4.9%) 868 (8.8%) 985 (10.0%) 531 (5.4%) 667 (6.8%) 1,132 (11.5%) 1,681 (17.0%) 674 (6.8%)	< 0.001
Living in rural areas	217 (16.7%)	996 (14.1%)	1,353 (13.7%)	0.034
Income quintile * 1 2 3 4 5	383 (29.5%) 311 (23.9%) 238 (18.3%) 208 (16.0%) 157 (12.1%)	1,672 (23.7%) 1,594 (22.6%) 1,306 (18.5%) 1,258 (17.9%) 1,199 (17.0%)	2,727 (27.6%) 2,256 (22.8%) 1,811 (18.3%) 1,584 (16.0%) 1,488 (15.1%)	< 0.001
Households and dwellings quintile* 1 2 3 4 5	154 (11.8%) 206 (15.8%) 247 (19.0%) 284 (21.8%) 396 (30.5%)	918 (13.0%) 1,172 (16.6%) 1,416 (20.1%) 1,481 (21.0%) 2,001 (28.4%)	1,359 (13.8%) 1,643 (16.6%) 1,868 (18.9%) 2,191 (22.2%) 2,738 (27.7%)	0.178
Racialized and newcomer quintile* 1 2 3 4 5	305 (23.5%) 252 (19.4%) 226 (17.4%) 264 (20.3%) 240 (18.5%)	1,606 (22.8%) 1,552 (22.0%) 1,371 (19.5%) 1,358 (19.3%) 1,101 (15.6%)	2,161 (21.9%) 2,025 (20.5%) 1,810 (18.3%) 1,866 (18.9%) 1,937 (19.6%)	< 0.001
Number of vaccine doses before PCR test ≤2 3 4 5 ≥ 6	1,185 (91.1%) 40 (3.1%) 38 (2.9%) 24 (1.8%) 13 (1%)	280 (3.9%) 2,985 (42.4%) 1,729 (24.5%) 1,124 (16.0%) 927 (13.1%)	2,722(27.5%) 3,744 (37.9%) 1,780 (18.0%) 1,028 (10.4%) 602 (6%)	< 0.001
Month of PCR test Jan-22 Feb-22 Mar-22 Apr-22 May-22 Jun-22 Jul-22 Aug-22 Sep-22 Oct-22 Nov-22 Dec-22 Jan-23 Feb-23 Mar-23 Apr-23 May-23 Jun-23 Jul-23 Aug-23 Sep-23 Oct-23 Nov-23 Dec-23 Jan-24 Feb-24 Mar-24 Apr-24 May-24 Jun-24 Jul-24 Aug-24 Sep-24 Oct-24 Nov-24 Dec-24 Jan-June 25	111 (8.5%) 60 (4.6%) 59 (4.5%) 61 (4.7%) 58 (4.5%) 52 (4.0%) 41 (3.2%) 35 (2.7%) 42 (3.2%) 53 (4.1%) 58 (4.5%) 44 (3.4%) 30 (2.3%) 46 (3.5%) 32 (2.5%) 31 (2.4%) 20 (1.5%) 24 (1.8%) 18 (1.4%) 19 (1.5%) 31 (2.4%) 30 (2.3%) 36 (2.8%) 27 (2.1%) 35 (2.7%) 22 (1.7%) 25 (1.9%) 25 (1.9%) 26 (2.0%) 19 (1.5%) 24 (1.8%) 21 (1.6%) 18 (1.4%) 11 (0.8%) 11 (0.8%) 8 (0.6%) 37 (2.8%)	709 (10.1%) 653 (9.3%) 655 (9.3%) 586 (8.3%) 522 (7.4%) 370 (5.3%) 231 (3.3%) 219 (3.1%) 241 (3.4%) 215 (3.1%) 219 (3.1%) 236 (3.3%) 262 (3.7%) 231 (3.3%) 242 (3.4%) 129 (1.8%) 70 (1.0%) 45 (0.6%) 34 (0.5%) 35 (0.5%) 28 (0.4%) 35 (0.5%) 71 (1.0%) 109 (1.5%) 167 (2.4%) 147 (2.1%) 127 (1.8%) 111 (1.6%) 54 (0.8%) 19 (0.3%) 20 (0.3%) 17 (0.2%) 16 (0.2%) 16 (0.2%) 18 (0.3%) 42 (0.6%) 141(2%)	275 (2.8%) 124 (1.3%) 145 (1.5%) 156 (1.6%) 141 (1.4%) 210 (2.1%) 307 (3.1%) 359 (3.6%) 350 (3.5%) 381 (3.9%) 370 (3.7%) 310 (3.1%) 305 (3.1%) 274 (2.8%) 317 (3.2%) 307 (3.1%) 294 (3.0%) 221 (2.2%) 230 (2.3%) 271 (2.7%) 263 (2.7%) 358 (3.6%) 324 (3.3%) 303 (3.1%) 277 (2.8%) 218 (2.2%) 209 (2.1%) 242 (2.4%) 300 (3.0%) 275 (2.8%) 308 (3.1%) 287 (2.9%) 263 (2.7%) 178 (1.8%) 154 (1.6%) 133 (1.3%) 440 4.4%)	< 0.001
Influenza vaccination	79 (6.1%)	2,508 (35.6%)	2,538 (25.7%)	< 0.001
Previous TIA/stroke	48 (3.7%)	304 (4.3%)	409 (4.1%)	0.568
Asthma	227 (17.5%)	1,331 (18.9%)	1,889 (19.1%)	0.356
Previous Cardiac ischemia	159 (12.2%)	1,182 (16.8%)	1,536 (15.5%)	< 0.001
CHF	170 (13.1%)	1,267 (18.0%)	1,504 (15.2%)	< 0.001
Chronic kidney disease	138 (10.6%)	1,144 (16.2%)	1,535 (15.5%)	< 0.001
COPD	438 (33.7%)	2,539 (36.0%)	3,604 (36.5%)	0.142
Dementia	54 (4.2%)	352 (5.0%)	413 (4.2%)	0.034
Diabetes	311 (23.9%)	2,325 (33.0%)	3,260 (33.0%)	< 0.001
Frailty score, mean ± SD	3.12 ± 5.32	3.24 ± 5.35	3.42 ± 5.31	0.031
Hypertension	731 (56.2%)	5,223 (74.1%)	6,792 (68.8%)	< 0.001
Inflammatory bowel disease	23 (1.8%)	103 (1.5%)	132 (1.3%)	0.423
Immunocompromised**	≤5 (≤0.4%)	68 (1.0%)	77 (0.8%)	0.044
Active liver disease or cirrhosis	61 (4.7%)	315 (4.5%)	461 (4.7%)	0.823
Charlson Index, mean ± SD	0.86 ± 1.24	1.01 ± 1.38	1.01 ± 1.36	0.002
Number of hospital visits in last 3 years, mean ± SD	1.75 ± 1.81	1.94 ± 1.71	1.95 ± 1.65	< 0.001
Number of general physician visits in last 3 years, mean ± SD	6.94 ± 8.39	8.16 ± 8.02	7.90 ± 7.88	< 0.001
Number of specialist visits in last 3 years, mean ± SD	9.76 ± 9.35	13.08 ± 10.06	12.78 ± 10.28	< 0.001

SD: Standard deviation * Missing < 0.3% **HIV infection, Organ transplant

**Table 3:** Absolute, seasonal and relative vaccine effectiveness (VE) for severe COVID-19 outcomes

Estimate	Absolute VE (95% CI)	Seasonal VE (95% CI)	Relative VE (95% CI)
Cohort 7–179 days post-vaccination vs. unvaccinated	Unadjusted: 42% (30%, 51%) Adjusted: 52% (42%, 60%)	Unadjusted: 34% (27%, 40%) Adjusted: 44% (37%, 50%)	Unadjusted: 33% (25%, 39%) Adjusted: 42% (35%, 49%)
Cohort ≥180 days post-vaccination vs. unvaccinated	Unadjusted: 13% (−3%, 27%) Adjusted: 16% (0%, 30%)		
Sensitivity analysis 7–179 days post-vaccination vs. unvaccinated	Unadjusted: 41% (29%, 51%) Adjusted: 49% (39%, 58%)	Unadjusted: 32% (25%, 38%) Adjusted: 49% (39%, 58%)	Unadjusted: 31% (23%, 37%) Adjusted: 42% (34%, 48%)
Sensitivity analysis ≥180 days post-vaccination vs. unvaccinated	Unadjusted: 15% (−1%, 28%) Adjusted: 12% (−4%, 26%)		

### Sensitivity analyses

**Fig. 2:**
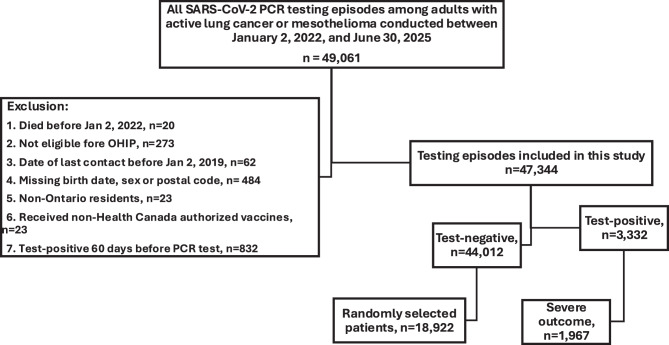
Study flowchart for sensitivity analysis

In sensitivity analyses, the 1,967 test-positive patients were compared with 18,922 randomly selected test-negative patients (Fig. 2). Patient characteristics for the sensitivity analysis are shown in Supplemental Table 3. In sensitivity analysis, we also compared patient characteristics among 1480 individuals who were unvaccinated at the time of SARS-CoV-2 testing, 7,975 patients who were tested 7–179 days after vaccination, and 11,434 who were tested ≥180 days post-vaccination (Supplemental Table 4). In sensitivity analysis, 3,397 (18.0%) test-negative patients and 623 (31.7%) test-positive patients died (*p* < 0.001). Hospitalization without death occurred in 10,612 (56.1%) test-negative patients and 1,344 (68.3%) test-positive patients (*p* < 0.001). In adjusted models, VE against severe COVID-19 was 49% (95% CI: 39%, 58%), declining to 12% (95% CI: −4%, 26%) among individuals ≥180 days after their most recent vaccine dose. Adjusted seasonal VE was 43% (95% CI: 36%, 49%), while the adjusted relative VE against severe COVID-19 was 42% (95% CI: 34%, 48%). Table 3 summarizes absolute, seasonal and relative VE estimates for severe outcome.

## Discussion

Our study demonstrated a moderate level of protection in the first six months following vaccination, with an estimated VE of 52% (95% CI: 42%, 60%) against severe COVID-19 among patients with active lung cancer. VE estimates remained relatively consistent across both primary and sensitivity analyses for seasonal and relative VE.

Notably, VE in patients with lung cancer was significantly lower than the VE estimates for severe COVID-19 in the general population. A recent study on Ontario’s general population estimated the COVID-19 VE to be 95% (95%CI: 87%, 98%) against severe COVID-19 outcomes 7–180 days after vaccination in the Omicron era [28]. The suboptimal VE against severe COVID-19 in our study is a critical finding, given that patients with lung cancer face the highest risk of COVID-19-associated mortality among patients with solid tumors, potentially due to reduced pulmonary function resulting from cancer or pre-existing conditions such as tobacco use or chronic obstructive pulmonary disease (COPD) [10]. Our finding of a moderate VE against severe COVID-19 outcomes among patients with lung cancer and mesothelioma aligns with prior evidence demonstrating reduced vaccine effectiveness in oncology and other immunocompromised populations compared with the general population. In a recent large population-based cohort study of cancer patients vaccinated during a period comparable to our cohort, the adjusted VE was 29.9% for preventing COVID-19-related hospitalization and 30.1% for ICU admission [29]. Unlike our study, that analysis included patients with a broad range of malignancies, did not assess mortality, and did not evaluate a composite outcome of hospitalization or death.

Another key finding of this study is the waning of VE in patients with lung cancer. Observational studies in the general population have demonstrated that VE against severe COVID-19 remains around 90% for over nine months post-vaccination [30]. In contrast, our study revealed a significant decline in VE beyond six months among patients with lung cancer, highlighting the critical need for booster doses in this high-risk population.

The reduced VE observed in patients with lung cancer compared to the general population may stem from several factors. Lung cancer compromises pulmonary immune responses, impairing both innate protection and the ability to mount an adequate immune response to vaccination, resulting in an increased risk of severe COVID-19 [31]. Chemotherapy, radiation, and immunotherapy may further suppress immune system function, resulting in lower VE [32, 33]. Timing of vaccination is also crucial- patients receiving vaccines shortly after chemotherapy may not mount a robust immune response [34]. Moreover, steroid use, common in lung cancer management for inflammation control, immune-related adverse events, symptom relief and palliative care, may further dampen vaccine efficacy. Other contributing factors include advanced age [35], a high burden of pulmonary and cardiovascular comorbidities [36–38], and negative effect of malnutritional and poor health status on immune responses [39, 40]. Given these biological and clinical factors, a lower VE against severe COVID-19 in lung cancer patients is expected, underscoring the need for alternative protective strategies.

The Omicron variant of SARS-CoV-2 seems to be associated with a lower risk of severe COVID-19 compared to previous variants. A recent study in the general population found that COVID-19 in the Omicron era is less likely to result in severe outcomes than during the Delta era. [41] This difference may be due to a reduced virulence associated with the Omicron SARS-CoV-2 variant or pre-existing immunity from prior infections or vaccination. However, the assumption that Omicron carries a lower mortality risk may not apply to patients with lung cancer. In the Delta era, the 28-day mortality rate among lung cancer patients with COVID-19 was 7.27% [10]. In the current study, this rate was 4.01% (242/6,037 patients). Despite this decline, mortality among hospitalized patients remained alarmingly high—45.8% in the Delta era and 52.07% in the Omicron era. These findings underscore the critical need to enhance preventive strategies and improve vaccination uptake in patients with lung cancer to reduce the risk of COVID-19-related hospitalization and death [10].

Our study has several limitations. First, we defined the outcome as COVID-19-associated all-cause hospitalization or death, and we could not determine the exact cause of hospitalization or mortality. Second, data on cancer treatment type and duration, which could influence VE and COVID-19 outcomes, were not available in our population-based dataset. This limitation is common in large-scale population-based studies. Third, the administrative databases did not include detailed symptom-level information or distinguish whether symptoms were attributable to underlying malignancy or SARS-CoV-2 infection. However, it can be reasonably inferred that nearly all tested individuals had clinical indications consistent with acute respiratory illness. Fourth, in calculating relative VE, individuals whose most recent COVID-19 vaccine dose was administered ≥180 days before testing were used as the reference group, representing those with waning vaccine-induced protection and some heterogeneity in residual immunity.

## Conclusions

Patients with lung cancer remain at high risk for severe COVID-19 outcomes, including elevated mortality rates following hospitalization and reduced VE in preventing severe disease. To mitigate this risk, a multifaceted approach is essential—optimizing comorbidity management, educating patients and caregivers, vaccinating close contacts, and advancing research into tailored vaccination strategies.

## Electronic supplementary material

Below is the link to the electronic supplementary material.

Supplementary Material 1

## Data Availability

Data are available from the authors upon reasonable request and with permission of ICES.
