# Supplementary material for: COVID-19 vaccine effectiveness in patients with lung cancer: challenges in the Omicron era
Source: BMC Infect Dis. 2026 Feb 6;26:692. doi: 10.1186/s12879-026-12741-9 (PMC13040916; doi:10.1186/s12879-026-12741-9)
Supplement: Supplementary file 1 [file 12879_2026_12741_MOESM1_ESM.docx]

# COVID-19 vaccine effectiveness in patients with lung cancer and mesothelioma in the Omicron era

Seyed M. Hosseini-Moghaddam, Frances A. Shepherd, Sarah Swayze, Jeffrey C. Kwong

Supplemental Table 1. Checklist of REporting of studies Conducted using Observational Routinely-collected health Data (RECORD) Statement 

|  | Item No | STROBE items | RECORD items | Reported |
| --- | --- | --- | --- | --- |
| Title and abstract | 1 | (a) Indicate the study's design with a commonly used term in the title or the abstract. (b) Provide in the abstract an informative and balanced summary of what was done and what was found. | (1.1) The type of data used should be specified in the title or abstract. When possible, the name of the databases used should be included. (1.2) If applicable, the geographic region and time frame within which the study took place should be reported in the title or abstract. (1.3) If linkage between databases was conducted for the study, this should be clearly stated in the title or abstract. | Abstract |
| Introduction |  |  |  |  |
| Background/ rationale | 2 | Explain the scientific background and rationale for the investigation being reported. |  | Introduction |
| Objectives | 3 | State specific objectives, including any prespecified hypotheses. |  | Introduction |
| Methods |  |  |  |  |
| Study design | 4 | Present key elements of study design early in the paper. |  | Methods |
| Setting | 5 | Describe the setting, locations, and relevant dates, including periods of recruitment, exposure, follow-up, and data collection. |  | Methods |
| Participants | 6 | (a) Give the eligibility criteria, and the sources and methods of selection of participants. Describe methods of follow-up.  (b) For matched studies, give matching criteria and number of exposed and unexposed. | (6.1) The methods of study population selection (such as codes or algorithms used to identify subjects) should be listed in detail. If this is not possible, an explanation should be provided. (6.2) Any validation studies of the codes or algorithms used to select the population should be referenced. If validation was conducted for this study and not published elsewhere, detailed methods and results should be provided.  (6.3) If the study involved linkage of databases, consider use of a flow diagram or other graphical display to demonstrate the data linkage process, including the number of individuals with linked data at each stage. | Methods, Appendix |
| Variables | 7 | Clearly define all outcomes, exposures, predictors, potential confounders, and effect modifiers. Give diagnostic criteria, if applicable. | (7.1) A complete list of codes and algorithms used to classify exposures, outcomes, confounders, and effect modifiers should be provided. If these cannot be reported, an explanation should be provided. | Methods, Appendix |
| Data sources/     measurement | 8 | For each variable of interest, give sources of data and details of methods of assessment (measurement). Describe comparability of assessment methods if there is more than one group. |  | Methods, Appendix |
| Bias | 9 | Describe any efforts to address potential sources of bias. |  | Methods, Discussion |
| Study size | 10 | Explain how the study size was arrived at. |  | Methods, Results |
| Quantitative variables | 11 | Explain how quantitative variables were handled in the analyses. If applicable, describe which groupings were chosen and why. |  | Methods |
| Statistical methods | 12 | (a) Describe all statistical methods, including those used to control for confounding. (b) Describe any methods used to examine subgroups and interactions. (c) Explain how missing data were addressed. (d) If applicable, explain how loss to follow-up was addressed. (e) Describe any sensitivity analyses. |  | Methods |
| Data access and cleaning methods |  | N/A | (12.1) Authors should describe the extent to which the investigators had access to the database population used to create the study population. (12.2) Authors should provide information on the data cleaning methods used in the study. | Methods |
| Linkage |  | N/A | (12.3) State whether the study included person-level, institutional-level, or other data linkage across two or more databases. The methods of linkage and methods of linkage quality evaluation should be provided. | Methods |
| Results |  |  |  |  |
| Participants | 13 | (a) Report numbers of individuals at each stage of study--e.g. numbers potentially eligible, examined for eligibility, confirmed eligible, included in the study, completing follow-up, and analyzed. (b) Give reasons for non-participation at each stage. (c) Consider use of a flow diagram. | (13.1) Describe in detail the selection of the persons included in the study (i.e., study population selection), including filtering based on data quality, data availability, and linkage. The selection of included persons can be described in the text and/or by means of the study flow diagram. | Methods, Results |
| Descriptive data | 14 | (a) Give characteristics of study participants (e.g. demographic, clinical, and social) and information on exposures and potential confounders. (b) Indicate number of participants with missing data for each variable of interest. (c) Summarize follow-up time (e.g. average and total amount). |  | Results |
| Outcome data | 15 | Report numbers of outcome events or summary measures over time. |  | Results |
| Main results | 16 | (a) Give unadjusted estimates and, if applicable, confounder-adjusted estimates and their precision (e.g. 95% confidence interval). Make clear which confounders were adjusted for and why they were included. (b) Report category boundaries when continuous variables were categorized. (c) If relevant, consider translating estimates of relative risk into absolute risk for a meaningful time period. |  | Results |
| Other analyses | 17 | Report other analyses done (e.g. analyses of subgroups and interactions, and sensitivity analyses). |  | Results |
| Key results | 18 | Summarize key results with reference to study objectives. |  | Discussion |
| Limitations | 19 | Discuss limitations of the study, taking into account sources of potential bias or imprecision. Discuss both direction and magnitude of any potential bias. | (19.1) Discuss the implications of using data that were not created or collected to answer the specific research question(s). Include discussion of misclassification bias, unmeasured confounding, missing data, and changing eligibility over time, as they pertain to the study being reported. | Discussion |
| Interpretation | 20 | Give a cautious overall interpretation of results considering objectives, limitations, multiplicity of analyses, results from similar studies, and other relevant evidence. |  | Discussion |
| Generalizability | 21 | Discuss the generalizability (external validity) of the study results. |  | Discussion |
| Other information |  |  |  |
| Funding | 22 | Give the source of funding and the role of the funders for the present study and, if applicable, for the original study on which the present article is based. |  | Acknowledgments & Funding |
| Accessibility of protocol, raw data, and programming code |  | N/A | (22.1) Authors should provide information on how to access any supplemental information such as the study protocol, raw data, or programming code. | Methods, Appendix |

Supplemental Table 2- Data sources used for comorbidities and medical conditions

| Medical Condition | Definition |
| --- | --- |
| Asthma (ICES cohort) | ## Asthma database was used to identify patients with asthma, based on 2 or more ambulatory care visits and/or 1 or more hospitalizations. ## OHIP ## OHIP diagnostic code: 493 ## CIHI-DAD ## ICD-9 diagnostic code: 493 ## ICD-10 diagnostic codes: J45, J46 |
| Chronic obstructive pulmonary disease (COPD) (ICES specific cohort) | COPD database was used to identify patients with COPD, based on 1 or more ambulatory care visits and/or 1 or more hospitalizations. Algorithm to identify COPD patients were only validated in those ages 35 and over.(2) OHIP OHIP diagnostic codes: 491, 492, 496 ## CIHI-DAD ICD-9 diagnostic codes: 491, 492, 496 ICD-10 diagnostic codes: J41, J42, J43, J44 |
| Diabetes (ICES cohort) | ODD was used to identify patients with diabetes, based on 2 OHIP diagnostic codes or 1 OHIP service code or 1 CIHI admission within 2 years.(3) OHIP OHIP diagnostic code: 250 OHIP service codes: Q040, K029, K030, K045, K046 CIHI-DAD, CIHI-SDS ICD-9 diagnostic code: 250 ICD-10 diagnostic codes: E10, E11, E13, E14 |
| Active cancer | i) Had any of the following treatments in the past 6 months: - Cancer surgery – see “SurgeryIn6Month_CCI_for Active Cancer concept.xlsx” CCI codes in DAD - Radiation -  using %getnacrs where source = Cancer and DX10CODE1 = Z510 - Chemotherapy -  using %getnacrs where source = Cancer and DX10CODE1 = Z511 or Z512 AND any evidence of cancer diagnosis in OCR prior to last treatment date (surgery, radiation and chemotherapy). II) If not I) (i.e., no recent treatment for cancer), if they had a recent diagnosis – cancer diagnosis in OCR within the past year before index |
| Chronic kidney disease (CKD) (DAD, NACRS, OHIP) | CKD diagnosis code in DAD, NACRS, OHIP in 5 years or At least 1 dialysis code in each of the 3 months prior to index Diagnosis and procedure codes found in concept dictionary OHIP OHIP diagnostic codes: 403, 585 CIHI NACRS, CIHI-DAD ICD-10 diagnostic codes: E102, E112, E132, E142, I12, I13, N08, N18, N19 |
| Patients who were on chronic dialysis (6) in the year before index date, identified as those with at least 2 of any of the following codes in OHIP, CIHI-DAD, or CIHI-SDS separated by at least 90 days, but less than 150 days OHIP OHIP service codes: R849, G323, G325, G326, G860, G862, G865 G863, G866, G330, G331, G332, G333, G861, G082, G083, G085, G090, G091, G092, G093, G094, G095, G096, G294, G295, G864, H540, H740 CIHI-DAD, CIHI-SDS CCI procedure codes: 5195, 6698 CCP procedure code: 1PZ21 |
| Advanced liver disease (Cirrhosis or Decompensated Cirrhosis) | See Lapointe-Shaw 2018 “Identifying cirrhosis…” Any history of: look back to start of data Cirrhosis Algorithm 9 2+ Physician Visit (dxcode 571) or 1+ Hospital Diagnosis CIRRHOSIS ICD-9 : 456.1, 571.2, 571.5 ICD-10: I85.9, I98.2, K70.3,K71.7, K74.6 Decompensated Cirrhosis Algorithm 5 1+ Physician Visit CIRRHOSIS (dxcode 571) and (1+ Hospital Diagnosis or 1+ Procedure) ICD-9: 456.0, 456.2, 572.2, 572.3, 572.4, 782.4, 789.5 ICD-10: I85.0, I86.4, I98.20, I98.3, K721, K729, K76.6, K76.7, R17, R18 CCI: 1.NA.13.BA-FA, 1.NA.13.BA-X7, 1.NA.13.BA-BD, 1.KQ.76GP-NR, 1.OT.52.HA CCP: 1006, 6691 OHIP: J057, Z591 |
| Immunocompromised (HIV, transplant, immunosuppressive therapy) | HIV (HIV) Transplant recipient (solid organ or stem cell) Solid organ transplant (CORR): For transplants before December 31, 2019: Use RECIPIENT_TREATMENT dataset in CORR library, and flag individuals where treatment_code=171 and treatment_date was before index date For transplants on/after January 1, 2020: Look for ICD-10 dx, CCI procedure, and OHIP feecodes from DAD, NACRS and OHIP for RENAL, LIVER, LUNG, HEART, PANCREAS, INTESTINE (see “All transplant types – 2021_01_19_JK.xlsx”) Use %dadsdsflags, %nacrsflags, %ohipflags Create a composite variable that includes all organs Allogenic/autologous bone marrow transplant (DAD, OHIP): Identify individuals with any of the following codes from database inception to index date: DAD: CCP (prcode) = 53.0 CCI (incode) = 1WY19, 1LZ19HHU7, 1LZ19HHU8 OHIP Feecode = Z426 Sickle cell anemia: Use %getdadsds to look for any hospitalization with any diagnosis code with : ICD9: 282.6 ICD10: D57.0-D57.2, D57.8 Other immune system disorders Any hospitalization, ED visit or physician billing (DAD, NACRS, OHIP; each from database inception) with the following codes: DAD/NACRS: ICD-9: 273.2, 279.0, 279.1 , 279.2, 279.3, 279.8, 279.9, 289.8, (NOTE: Only 4-digit ICD9 codes found) ICD-10: D80, D81, D82, D83, D84, D89 OHIP: dxcode = 279 Immunosuppressive therapy (see DIN list: “immunomeds_antineo_25JAN2021.xlsx”) >30 days (total days supplied) of oral corticosteroid in the 6 months before index Receipt of other immunocompromising drug (including antineoplastics) in the 6 months before index |
| Inflammatory Bowel Disease | OCCC cohort definition |
| Dementia/Frailty | Dementia (ICES cohort): 1 hospitalization for dementia and/or 3 ambulatory visits for dementia, each separated by at least 30 days, within 2 years and/or 1 prescription from ODB (9) OHIP OHIP diagnostic codes: 290, 331 CIHI-DAD, CIHI-SDS ICD-9 diagnostic codes: 0461, 290.0, 290.1, 290.2, 290.3, 290.4, 294, 331.0, 331.1, 331.5 ICD-10 diagnostic codes: F00, F01, F02, F03, G30 ODB 1 prescription for a cholinesterase inhibitor Frailty: Hospital Frailty Risk Score - “Summarized” frailty score – Pull all DAD hospitalizations in the 5-years before index, and use %hospfrailty macro to determine “summarized” frailty score per IKN. (Gilbert 2018, Lancet) NOTE: Codes used in this risk score also include frailty related conditions such as dementia and other chronic conditions that are reported separately. |
| Hypertension | HYPER database |
| History of congestive heart failure (CHF) (ICES cohort) | CHF database was used to identify patients with CHF, based on 1 CIHI NACRS, CIHI-DAD, CIHI-SDS, or OHIP claim and a second claim (from either) in 1 year. The CHF database is limited to those 40 years of age or older.(11) OHIP OHIP diagnostic code: 428 CIHI-DAD, CIHI-SDS ICD-9 diagnostic code: 428 ICD-10 diagnostic codes: I500, I501, I509 |
| History of TIA or Acute Ischemic Stroke | Transient Ischemic Attack: CIHI-DAD and CIHI-NACRS were used to identify patients with a history of a transient ischemic attack, based on at least 1 hospitalization or ED visit with a diagnosis coded with one of the following codes: ICD-9 diagnostic codes: 435, 3623 ICD-10 diagnostic codes: G450, G451, G452, G453, G458, G459, H340 Acute Ischemic Stroke (12): CIHI-DAD was used to identify patients with a history of acute ischemic stroke, based on at least 1 hospitalization with a main diagnosis coded with one of the following codes: ICD-9 diagnostic codes: 43301, 43311 43321 43331 43381 43391 434, 436 ICD-10 diagnostic codes: I63, I64, H34.1 |
| History of cardiac ischemia | Cardiac ischemic disease (DAD, SDS): Any comorbidty in the past 5 years (DAD, any diagnosis field) or history of procedure in past 20 years (DAD, SDS): Comorbidity (DAD, any dx, 5-year lookback): Angina: ICD-10 diagnostic codes: I20 ICD-9: 413 Chronic Ischemic Heart Disease: ICD-10 diagnostic codes: I25 ICD-9: 4140, 4148, 4149  Myocardial infarction: ICD-10 diagnostic codes: I21, I22 ICD-9: 410, 411, 412  Procedure (DAD & SDS, 20 year lookback): Coronary Artery Bypass Grafting: CCI procedure codes: 1IJ76 CCP procedure codes: 481  Percutaneous Coronary Intervention: CCI procedure codes: 1IJ50, 1IJ5, 1IJ57 CCP procedure codes: 4802, 4803 |
| Influenza vaccination | OHIP: Use %getohip (nonlab) to search for any of the following feecodes from the most recent October 1 up to 14 days before index date: G590, G591, G592, Q130, Q590, Q690, Q691 ODB: Use %getodb to search for any of the following DINs from the most recent October 1 up to 14 days before index date: 2019/20: 02420643, 02420783, 02432730, 02473283 2020/21: 02420643, 02420783, 02432730, 02445646, 02494248, 09857645, 09857646 **NOTE: To include DINs from <65, change default parameter to age 65=F. |
| COVID-19 vaccination | COVaxON |
| Death | RPDB |

Supplemental Table 3-Comparison of patients with severe COVID-19 and randomly selected test negative

patients

| Variable | Randomly selected  test-negative patients N= 18,922 | Patients with severe COVID-19, N= 1,967 | P-Value |
| --- | --- | --- | --- |
| Age (year), mean ± SD | 70.37 ± 10.05 | 71.48 ± 10.09 | <0.001 |
| Sex F M | 9,439 (49.9%) 9,483 (50.1%) | 915 (46.5%) 1,052 (53.5%) | 0.004 |
| PHU region* Central East Central West Durham Eastern Northern Ottawa Peel South West Toronto York | 1,783 (9.4%) 3,498 (18.5%) 904 (4.8%) 1,797 (9.5%) 1,846 (9.8%) 1,086 (5.7%) 1,166 (6.2%) 2,271 (12.0%) 3,044 (16.1%) 1,276 (6.7%) | 148 (7.5%) 374 (19.0%) 73 (3.7%) 149 (7.6%) 197 (10.0%) 119 (6.0%) 146 (7.4%) 2,271 (12.0%) 354 (18.0%) 138 (7.0%) | <0.001 |
| Living in rural areas | 2,749 (14.5%) | 221 (11.2%) | <0.001 |
| Neighborhood income quintile * 1 2 3 4 5 | 4,888 (25.8%) 4,280 (22.6%) 3,485 (18.4%) 3,251 (17.2%) 2,984 (15.8%) | 535 (27.2%) 445 (22.6%) 365 (18.6%) 314 (16.0%) 305 (15.5%) | 0.700 |
| Vaccine doses prior to PCR test 0 1 2 3 4 5+ | 1,156 (6.1%) 198 (1.0%) 2,796 (14.8%) 6,993 (37.0%) 3,710 (19.6%) 4,069 (21.5%) | 166 (8.4%) 27 (1.4%) 334 (17.0%) 697 (35.4%) 393 (20.0%) 350 (17.8%) | <.001 |
| Vaccination Status at PCR test date Unvaccinated 7-179 days post- vaccination ≥180 days post-vaccination | 1,294 (6.8%) 7,391 (39.1%) 10,237 (54.1%) | 178 (9.0%) 596 (30.3%) 1,193 (60.7%) | <.001 |
| Month of SARS-CoV-2 PCR test Jan-22 Feb-22 Mar-22 Apr-22 May-22 Jun-22 Jul-22 Aug-22 Sep-22 Oct-22 Nov-22 Dec-22 Jan-23 Feb-23 Mar-23 Apr-23 May-23 June 23 Jul-23 Aug-23 Sep-23 Oct-23 Nov-23 Dec-23 Jan-24 Feb-24 Mar-24 Apr-24 May-24 Jun-24 Jul-24 Aug-24 Sep-24 Oct-24 Nov-24 Dec-24 Jan- June 25 | 1,049 (5.5%) 881 (4.7%) 854 (4.5%) 828 (4.4%) 803 (4.2%) 669 (3.5%) 560 (3.0%) 591 (3.1%) 591 (3.1%) 626 (3.3%) 638 (3.4%) 559 (3.0%) 548 (2.9%) 539 (2.8%) 589 (3.1%) 479 (2.5%) 416 (2.2%) 321 (1.7%) 305 (1.6%) 336 (1.8%) 345 (1.8%) 424 (2.2%) 415 (2.2%) 409 (2.2%) 497 (2.6%) 417 (2.2%) 417 (2.2%) 415 (2.2%) 426 (2.3%) 319 (1.7%) 368 (1.9%) 370 (2.0%) 341 (1.8%) 238 (1.3%) 210 (1.1%) 224 (1.2%) 905 (4.7%) | 1,049 (5.5%) 63 (3.2%) 50 (2.5%) 117 (5.9%) 81 (4.1%) 32 (1.6%) 72 (3.7%) 66 (3.4%) 80 (4.1%) 102 (5.2%) 85 (4.3%) 91 (4.6%) 82 (4.2%) 54 (2.7%) 68 (3.5%) 38 (1.9%) 26 (1.3%) 14 (0.7%) 17 (0.9%) 24 (1.2%) 38 (1.9%) 62 (3.2%) 72 (3.7%) 79 (4.0%) 75 (3.8%) 24 (1.2%) 9 (0.5%) 12 (0.6%) 16 (0.8%) 19 (1.0%) 32 (1.6%) 36 (1.8%) 37 (1.9%) 36 (1.8%) 30 (1.5%) 25 (1.3%) 44 (2.2%) | <.001 |
| Influenza vaccination, 2 recent seasons | 5,328 (28.2%) | 560 (28.5%) | 0.770 |
| Comorbidities Previous TIA/stroke Asthma Previous Cardiac ischemia Congestive heart failure Chronic kidney disease COPD Dementia Diabetes mellitus Hypertension Immunocompromised ** Liver disease or cirrhosis | 844 (4.5%) 3,616 (19.1%) 3,011 (15.9%) 2,973 (15.7%) 2,903 (15.3%) 6,815 (36.0%) 855 (4.5%) 5,955 (31.5%) 13,111 (69.3%) 155 (0.8%) 876 (4.6%) | 104 (5.3%) 397 (20.2%) 354 (18.0%) 401 (20.4%) 382 (19.4%) 769 (39.1%) 117 (5.9%) 713 (36.2%) 1,449 (73.7%) 21 (1.1%) 106 (5.4%) | 0.094 0.250 0.017 <0.001 <0.001 0.007 0.004 <0.001 <0.001 0.251 0.130 |
| Frailty score, Mean ± SD | 3.84 ± 5.58 | 5.59 ± 6.79 | <0.001 |
| Charlson-Deyo Comorbidity index, Mean ± SD | 1.03 ± 1.37 | 1.24 ± 1.48 | <0.001 |

COPD: Chronic obstructive pulmonary disease, SD: standard deviation

* Missing <0.3%

**HIV infection, Organ transplant

Supplemental Table 4- Comparison of unvaccinated during the season and individuals who were tested 7-179 and ≥ 180 days post- vaccination

| Variable | Unvaccinated N=1,480 | 7-179 days post-vaccination N=7,975 | ≥180 days post- vaccination, N=N=11,434 | P-value |
| --- | --- | --- | --- | --- |
| Age (year), mean ± SD | 66.71 ± 10.66 | 72.67 ± 9.27 | 69.43 ± 10.19 | <0.001 |
| Sex F M | 699 (47.2%) 781 (52.8%) | 3,986 (50.0%) 3,989 (50.0%) | 5,669 (49.6%) 5,765 (50.4%) | 0.151 |
| PHU region* Central East Central West Durham Eastern Northern Ottawa Peel South West Toronto York | 1,189 (9.2%) 2,441 (18.9%) 599 (4.6%) 1,141 (8.8%) 1,268 (9.8%) 684 (5.3%) 876 (6.8%) 1,499 (11.6%) 2,172 (16.8%) 885 (6.9%) | 741 (9.3%) 1,447 (18.1%) 376 (4.7%) 798 (10.0%) 763 (9.6%) 508 (6.4%) 446 (5.6%) 1,026 (12.9%) 1,217 (15.3%) 536 (6.7%) | 1,047 (9.2%) 2,154 (18.8%) 535 (4.7%) 1,009 (8.8%) 1,140 (10.0%) 629 (5.5%) 771 (6.7%) 1,294 (11.3%) 1,935 (16.9%) 781 (6.8%) | <0.001 |
| Living in rural areas | 244 (16.5%) | 1,143 (14.3%) | 1,576 (13.8%) | 0.035 |
| Households and dwellings quintile*  1 2 3 4 5 | 179 (12.1%) 277 (18.7%) 259 (17.5%) 302 (20.4%) 449 (30.3%) | 1,455 (18.2%) 1,558 (19.5%) 1,587 (19.9%) 1,575 (19.7%) 1,732 (21.7%) | 1,626 (14.2%) 2,059 (18.0%) 2,249 (19.7%) 2,401 (21.0%) 3,001 (26.2%) | <0.001 |
| Racialized and newcomer quintile* 1 2 3 4 5 | 355 (24.1%) 273 (18.5%) 264 (17.9%) 299 (20.3%) 266 (18.1%) | 1,855 (23.2%) 1,792 (22.4%) 1,545 (19.3%) 1,501 (18.8%) 1,226 (15.3%) | 2,523 (22.1%) 2,321 (20.3%) 2,079 (18.2%) 2,169 (19.0%) 2,242 (19.6%) | <0.001 |
| Number of vaccine doses before PCR test 0 1 2 3 4 ≥5 | 1,323 (89.4%) <=5 (<=0.3%) <=5 (<=0.2%) 54 (3.6%) 51 (3.4%) 44 (3.0%) | 0 (0.0%) 47 (0.6%) 244 (3.1%) 3,385 (42.4%) 1,967 (24.7%) 2,332 (29.2%) | 0 (0.0%) 168 (1.5%) 2,878 (25.2%) 4,275 (37.4%) 2,090 (18.3%) 2,023 (17.7%) | <0.001 |
| Month of PCR test Jan-22 Feb-22 Mar-22 Apr-22 May-22 Jun-22 Jul-22 Aug-22 Sep-22 Oct-22 Nov-22 Dec-22 Jan-23 Feb-23 Mar-23 Apr-23 May-23 Jun-23 Jul-23 Aug-23 Sep-23 Oct-23 Nov-23 Dec-23 Jan-24 Feb-24 Mar-24 Apr-24 May-24 Jun-24 Jul-24 Aug-24 Sep-24 Oct-24 Nov-24 Dec-24 Jan- June 25 | 123 (8.3%) 69 (4.7%) 55 (3.7%) 81 (5.5%) 57 (3.9%) 58 (3.9%) 43 (2.9%) 38 (2.6%) 48 (3.2%) 57 (3.9%) 72 (4.9%) 49 (3.3%) 38 (2.6%) 52 (3.5%) 39 (2.6%) 32 (2.2%) 21 (1.4%) 22 (1.5%) 19 (1.3%) 24 (1.6%) 35 (2.4%) 36 (2.4%) 38 (2.6%) 30 (2.0%) 47 (3.2%) 30 (2.0%) 23 (1.6%) 35 (2.4%) 23 (1.6%) 16 (1.1%) 28 (1.9%) 28 (1.9%) 21 (1.4%) 14 (0.9%) 12 (0.8%) 14 (0.9%) 52 (3.5%) | 806 (10.1%) 723 (9.1%) 709 (8.9%) 703 (8.8%) 625 (7.8%) 430 (5.4%) 251 (3.1%) 240 (3.0%) 261 (3.3%) 236 (3.0%) 231 (2.9%) 264 (3.3%) 269 (3.4%) 243 (3.0%) 265 (3.3%) 145 (1.8%) 72 (0.9%) 38 (0.5%) 42 (0.5%) 41 (0.5%) 34 (0.4%) 36 (0.5%) 89 (1.1%) 142 (1.8%) 183 (2.3%) 165 (2.1%) 149 (1.9%) 123 (1.5%) 65 (0.8%) 27 (0.3%) 20 (0.3%) 16 (0.2%) 22 (0.3%) 22 (0.3%) 29 (0.4%) 48 (0.6%) 208 (2.6%) | 275 (2.4%) 154 (1.3%) 138 (1.2%) 193 (1.7%) 155 (1.4%) 216 (1.9%) 312 (2.7%) 404 (3.5%) 399 (3.5%) 424 (3.7%) 414 (3.6%) 353 (3.1%) 318 (2.8%) 310 (2.7%) 355 (3.1%) 333 (2.9%) 357 (3.1%) 257 (2.2%) 252 (2.2%) 277 (2.4%) 314 (2.7%) 399 (3.5%) 382 (3.3%) 354 (3.1%) 318 (2.8%) 259 (2.3%) 250 (2.2%) 275 (2.4%) 349 (3.1%) 328 (2.9%) 340 (3.0%) 339 (3.0%) 330 (2.9%) 248 (2.2%) 196 (1.7%) 179 (1.6%) 678 (5.9%) | <0.001 |
| Influenza vaccination | 95 (6.4%) | 2,899 (36.4%) | 2,899 (25.4%) | <0.001 |
| Previous TIA/stroke | 63 (4.3%) | 376 (4.7%) | 510 (4.5%) | 0.606 |
| Asthma | 258 (17.4%) | 1,540 (19.3%) | 2,215 (19.4%) | 0.196 |
| Previous Cardiac ischemia | 195 (13.2%) | 1,343 (16.8%) | 1,850 (16.2%) | 0.002 |
| CHF | 198 (13.4%) | 1,408 (17.7%) | 1,786 (15.6%) | <0.001 |
| Chronic kidney disease | 155 (10.5%) | 1,277 (16.0%) | 1,836 (16.1%) | <0.001 |
| COPD | 500 (33.8%) | 2,930 (36.7%) | 4,166 (36.4%) | 0.092 |
| Dementia | 59 (4.0%) | 429 (5.4%) | 480 (4.2%) | <0.001 |
| Diabetes | 352 (23.8%) | 2,567 (32.2%) | 3,747 (32.8%) | <0.001 |
| Frailty score, mean ± SD | 3.84 ± 5.66 | 3.78 ± 5.68 | 4.19 ± 5.73 | <0.001 |
| Hypertension | 827 (55.9%) | 5,874 (73.7%) | 7,850 (68.7%) | <0.001 |
| Inflammatory bowel disease | 22 (1.5%) | 120 (1.5%) | 153 (1.3%) | 0.607 |
| Immunocompromised** | 9 (0.6%) | 70 (0.9%) | 94 (0.8%) | 0.572 |
| Active liver disease or cirrhosis | 65 (4.4%) | 352 (4.4%) | 557 (4.9%) | 0.29 |
| Charlson Index, mean ± SD | 0.92 ± 1.31 | 1.05 ± 1.39 | 1.07 ± 1.39 | 0.001 |
| Number of hospital episodes of care in last 3 years, mean ± SD | 2.03 ± 1.85 | 2.15 ± 1.71 | 2.29 ± 1.78 | <0.001 |
| Number of general physician visits in last 3 years, mean ± SD | 7.46 ± 9.41 | 8.57 ± 8.45 | 8.43 ± 8.26 | <0.001 |
| Number of specialist visits in last 3 years, mean ± SD | 11.08 ± 10.00 | 13.88 ± 10.07 | 14.07 ± 10.82 | <0.001 |

SD: Standard deviation

* Missing <1.5%

**HIV infection, Organ transplant
